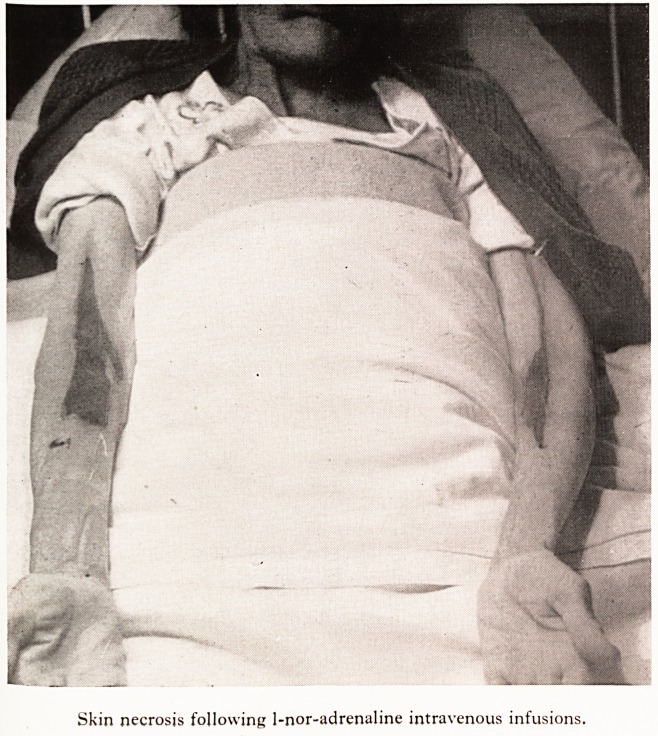# Supportive Treatment of Acute Cor Pulmonale Due to Massive Pulmonary Embolism

**Published:** 1958-04

**Authors:** E. A. W. Houghton

**Affiliations:** Formerly Medical Registrar, Southmead Hospital, Bristol


### SUPPORTIVE TREATMENT OF ACUTE COR PULMONALE DUE TO MASSIVE PULMONARY EMBOLISM

E. A. W. HOUGHTON, M.B., CH.B.

*Formerly Medical Registrar, Southmead Hospital, Bristol*

Pulmonary thrombo-embolism may cause a variety of clinical features. Generally, however, cases fall into three main groups; (1) those in which no immediate symptoms occur, but who may develop subacute cor pulmonale if embolism is recurrent; (2) those in which demonstrable pulmonary infarction occurs with absent or minimal circulatory signs, and (3) those cases in which there is an acute and profound circulatory disturbance, right ventricular failure predominating. The latter constitutes the syndrome of acute cor pulmonale.

The usual clinical features of acute cor pulmonale are pain or a sense of oppression in the centre of the chest, peripheral cyanosis or pallor, hypotension, raised jugular venous pressure and signs related to right ventricular and pulmonary artery dilatation. Typical electrocardiographic changes occur.

While pulmonary embolism is a common condition, the proportion of cases in which cor pulmonale occurs is small. Short (1952) in his survey of 113 cases, classified 16 as "massive", and among 34 cases seen by the present author in a period of a year 9 were in this category. In an acute hospital of 500 beds, 10 fatal cases are to be expected each year (Marks *et al.*, 1954).

Attention in recent years has been focused upon the problem of the prevention of peripheral venous thrombosis, particularly in the post-operative period. There has been a relative neglect of the management of the emergency of massive embolism. This may be partly due to an attitude of pessimism as to the outcome and the fact that in most hospitals the initial management is often in the hands of junior medical staff inexperienced in the treatment of this condition.

Treatment is primarily supportive. Measures advocated include analgesics, vagolytic agents, antispasmodics, myocardial stimulants and more recently pressor agents particularly l.nor-adrenaline. Anticoagulant therapy and possibly venous ligation may be considered as secondary measures to prevent further embolism.

The following case is reported as it illustrates many of the practical problems of management, and the success of vigorous action towards maintaining a normal blood pressure.

### CASE REPORT

**A woman aged 68 years** was admitted to Southmead Hospital, Bristol, on 24th November, 1953, following a haematemesis. She was known to have a chronic duodenal ulcer and had had several haematemeses before. On examination on this occasion a mass was palpable in the left iliac fossa. After cessation of the alimentary haemorrhage, sigmoidoscopy was performed, but only the lower 10 cm. of bowel was visible owing to spasm. A "blind" biopsy was taken which was reported as showing "columnar cell adenocarcinoma". Operation was advised but refused by the patient. A barium enema examination was unhelpful as the enema was not retained.

The patient was readmitted six months later on account of left-sided abdominal pain and vomiting. On 5th May, 1954, a laparotomy was performed by Mr. J. A. Pocock and a mass was found to be present in the sigmoid colon. A transverse colostomy was fashioned and the wound closed. On the eight post-operative day the patient was being wheeled along the ward when she suddenly experienced severe upper abdominal pain, tightness in the chest, and breathlessness. Examination revealed that she was cyanosed and shocked. The pulse was 130 per min., and systolic blood pressure 70 mm.Hg. The neck veins were engorged and pulsating. There was increased pulsation over the precordium to the left of the sternum at the level of the second and third interspaces. Breath sounds were reduced at the lung bases. There were no clinical signs of venous thrombosis in the legs. A clinical diagnosis of massive pulmonary embolism was made which was supported by an electrocardiogram. This showed high 'P' waves, a 'Q' wave in lead III, partial right bundle branch block, flattened 'T' waves and depression of the S-T segment.

Pethidine 100 mg. and methylamphetamine ("methedrine") 30 mg. were given intramuscularly, heparin 10,000 international units (77 mg.) intravenously and ethyl biscoumacetate ("tromexan") 900 mg. orally. Although there was slight relief of pain, the hypotension persisted and so, an hour after the onset, an intravenous infusion of 5 per cent dextrose with 4 ml. of 1:1000 l.nor-adrenaline per 500 ml. was commenced. This gave a concentration of 8 $\mu$g. per ml. There was an immediate response, the blood pressure rising to 130/100 mm.Hg. By regulating the speed of the infusion to 15–20 drops per minute the blood pressure was maintained at 110/90 mm.Hg. Papaverine ½ gr. (30 mg.) was also given intramuscularly. The serum prothrombin level was kept between 30 per cent and 60 per cent of normal by varying the dose of the anticoagulant.

Recurrent chest pain was a distressing feature during the following three days. This did not respond to conventional doses of pethidine or amidone, but rapid temporary relief followed introduction of 2–5 ml. of 2 per cent procaine hydrochloride into the infusion. Considerable difficulty was experienced in maintaining the l.nor-adrenaline infusion, for whenever it was slowed down appreciably the blood pressure fell and the patient lapsed into coma. Following leakage into the tissues at the original infusion sites in the anticubital fossae there was considerable local oedema, pain and tenderness. This was followed by discolouration of the skin which eventually sloughed. Skin loss was made good by skin grafting at a later date. Ouabain was given in doses of 1 mg. intravenously at six-hourly intervals during the first 24 hours but produced no noticeable improvement in the pulse or the patient's general condition.

During the fifth day of treatment it was found possible gradually to reduce the rate of the l.nor-adrenaline infusion without the blood pressure falling, and it was eventually discontinued on the sixth day.

Three weeks after the embolic incident the patient was ambulant and symptom-free apart from some breathlessness on exertion. The electrocardiogram gradually returned to normal, and the venous pressure, as judged by the neck veins, gradually fell. The heart remained clinically enlarged for several months but its size eventually returned to within normal limits. No definite signs of pulmonary infarction appeared in the chest clinically or radiologically. On the 28th July, 1954, the second stage of the sigmoid colon resection was carried out and was uneventful. Microscopical examination of the operation specimen revealed no sign of neoplasia, the appearances being consistent with diverticulitis.

The patient has continued in reasonably good health, apart from symptoms attributable to her peptic ulcer, and has not experienced any further symptoms referable to the cardiovascular system.

### DISCUSSION

Diagnosis in this case was not difficult as the symptoms and signs were typical. However, similar peripheral circulatory failure may be produced by other conditions, including myocardial infarction, concealed haemorrhage, supra-renal haemorrhage and severe septicaemia. Attention to the cardiac signs and the electrocardiogram will usually produce sufficient evidence of right ventricular failure or 'strain' for a diagnosis of acute cor pulmonale to be made.

The exact mechanism of the circulatory changes commonly seen is not perfectly understood. Experiments in animals in which the pulmonary arteries have been occluded in varying degree by ligature or by artificial emboli, have shown that it is necessary to obstruct 60–85 per cent of the total cross-section before the systolic blood pressure falls or before signs of right ventricular failure can be detected (Gibbon *et al.*, 1932). The term "right ventricular failure" is a misnomer, as the ventricle shows forceful action in its attempt to maintain a head of pressure so as to overcome the obstruction. At autopsy the chamber is usually engorged and the pulmonary artery dilated; when these features are not present vaso-motor shock, by reducing the return of blood to the right heart is probably responsible.

Fatal cases have been reported in which a relatively small embolism was present at autopsy, occupying only a small proportion of the pulmonary artery (Fowler and Bollinger, 1954). Reflex coronary artery spasm may play an important part in such cases. Attempts to prove this by animal experiments have been inconclusive, however. De Takats *et al.* (1939) found that atropine and papaverine apparently had a protective effect in dogs, but Malinow *et al.* (1946) state that bilateral vagotomy fails to influence the electrocardiographic changes. However, atropine and/or papaverine are still recommended in the standard textbooks (Brooks, 1952; Wood, 1956). In the case described papaverine did appear to relieve pain, but was accompanied by a fall of

PLATE I

Lead II of Electrocardiogram: (*a*) Three hours after massive pulmonary embolism.
(*b*) Three months later.

Skin necrosis following l-nor-adrenaline intravenous infusions.

blood pressure on one occasion when ½ gr. (30 mg.) was given intravenously. The relief of pain by procaine hydrochloride was first noticed when 2 ml. of 2 per cent solution was given intravenously to relieve venospasm when the intravenous infusion was behaving erratically. Its use has not been previously recorded in the literature for this specific purpose.

Churchill (1934) in his classic description of massive pulmonary embolism emphasized the potential harm of venesection which has been advocated in order to "take the load off the right ventricle". Wood (1947) has pointed out that the raised venous pressure is beneficial. For this reason digitalis is contra-indicated, while a strophanthin preparation, such as ouabain, has theoretical advantages as it is said to stimulate the myocardium without lowering the venous pressure. Rapidity of action is also in its favour, and ouabain in particular appears to exert a pressor effect in full therapeutic dosage (McMichael, 1950).

There is evidence that anticoagulant drugs will reduce the likelihood of further embolism (Barker *et al.*, 1945, Cosgriff, 1950), although fatal embolism may occur during anticoagulant treatment (Farris, 1954). A further reason for their use is that they prevent the formation of propagation clot beyond the impacted embolus. In this connection attention should be drawn to the work of Cummine and Lyons (1948) and Dew (1953) who suggest that primary thrombosis of the pulmonary artery is more common than is usually recognized. Burt (1954) reports that propagation clot is infrequently found in autopsy cases when anticoagulants have been used but she records having seen it in fatal cases when anticoagulants have not been employed.

Venous ligation as a prophylactic measure against further embolism is losing popularity (Farris, 1954); it is a gamble and late sequelae may be disabling if the inferior vena cava is tied (Shea *et al.*, 1951). This operation also has a significant mortality (Riddell and Kirtley, 1952), also it has been shown that the incidence of fatal embolism is not reduced by either prophylactic or therapeutic vein ligation (Lillie *et al.*, 1949; Kirby and Fitts, 1950).

The most important aspect of treatment in this case was undoubtedly the action of l.nor-adrenaline in raising and maintaining the blood pressure. This substance, which is normally present in the adrenal medulla and at the synapses of postganglionic sympathetic nerves has been widely used in a variety of hypotensive states since its introduction for this purpose by Goldenberg *et al.* (1949). Its use in acute cor pulmonale has been reported by Wolff (1954), de Swiet (1955) and Sibthorpe (1955). The mechanism of the beneficial action in such cases probably lies in the improved coronary circulation caused by active vasodilatation of the coronary arteries and by raised aortic pressure (von Euler, 1955). The concentration of the infusion used depends upon the individual response. de Swiet found that a concentration of 64 ml. of 1:1,000 solution per litre was necessary in his case so as to avoid risk of overloading the circulation with too large a volume of dextrose solution. Therapy must be continued until the balance of ventricular input and output is restored. Presumably this becomes possible through packing of the thrombus against the vessel wall and the gradual relaxation of any arterial spasm which may be present, together with improved function of the myocardium. The ganglion-blocking effect of l.nor-adrenaline which has been described by Burn (1956) and others, and recently emphasised by Mushin (1957), was not evident in this case when the drug was discontinued. During the long period of l.nor-adrenaline "dependence" there was ample evidence of a central cause for hypotension without incriminating the drug. However, the precaution was taken of gradually weaning the patient from the nor-adrenaline when her general condition had improved.

Skin necrosis following intravenous administration of l.nor-adrenaline has been reported by Greenwald *et al.* (1952), Bergmann (1953) and Humphreys *et al.* (1955). The latter authors discuss the possible causes in detail, and conclude that impaired peripheral circulation and a high local concentration of the drug is responsible. They advocate percutaneous administration into a large vein together with frequent introduction of papaverine and procaine into the vein to relieve local spasm. All these details were employed in this case but skin necrosis occurred. Constant vigilance and an awareness of the possibility of the complication are probably the most important factors in prevention.

SUMMARY

The treatment is reviewed of acute cor pulmonale due to massive pulmonary thrombo-embolism.

A case is described in which the use of a pressor agent, l.nor-adrenaline, appeared to play a prominent part in the successful outcome.

Early recognition of the circulatory features of this condition and its differentiation from other causes of shock is essential. The meticulous maintenance of normotension is the basis of treatment at the present time.

Further experimental and clinical research is needed in this subject.

ACKNOWLEDGMENTS

I am indebted to Mr. J. A. Pocock and Dr. H. J. Orr-Ewing for their permission to publish details of this case.

### REFERENCES

Barker, N. W., Cromer, H. E., Hurn, M., Waugh, J. M. (1945). *Surgery*, **17**, 207.
Bergmann, H. (1953). *Int. J. Anaes.*, **I**, 29.
Brooks, W. D. W. (1952). *Diseases of the Chest*, Marshall and Perry, Butterworth, London.
Burn, J. H. (1956). *Brit. J. Anaes.*, **28**, 466.
Burt, C. C. (1954). *Edinburgh Med. J.*, **61**, 273.
Churchill, E. D. (1934). *Surg. Gynec. Obstet.*, **59**, 513.
Cosgriff, S. W. (1950). *J. Amer. med. Ass.*, **143**, 870.
Cummine, H., Lyons, R. N. (1948). *Brit. J. Surg.*, **35**, 337.
Dew, H. (1953). *Ann. roy. Coll. Surg. Engl.*, **38**, 30.
von Euler, U. S. (1955). *Lancet*, **ii**, 151.
Farris, J. M. (1954). *Surg. Clin. N. Amer.*, **34**, 1271.
Fowler, E. F., Bollinger, J. A. (1954). *Surgery*, **36**, 650.
Gibbon, J. H., Hopkinson, M., Churchill, E. D. (1932). *J. Clin. Invest.*, **II**, 543.
Goldenberg, M., Apgar, V., Deterling, R., Pines, K. L. (1949). *J. Amer. med. Ass.*, **140**, 776.
Greenwald, H. P., Gootnick, A., Luger, N. M., King, J. A. (1953). *New Engl. J. Med.*, **246**, 252.
Lillie, R. H., Buwton, R. W., Duff, I. F. (1949). *Arch. Surg.*, **59**, 609.
Malinow, M. R., Katz, L. N., Kondo, B. (1946). *Amer. Heart. J.*, **31**, 702.
Marks, J., Truscott, B. B., Withycombe, J. F. R. (1954). *Lancet*, **ii**, 787.
McMichael, J. (1950). *Pharmacology of the Failing Human Heart*, Blackwell, Oxford.
Mushin, W. W. (1957). *Brit. Med. J.*, **i**, 1302.
Riddell, D. H., Kirtley, J. A. Jr., (1952). *J. Tenness. M.A.*, **45**, 347.
Shea, P. C., Robertson, R. L. (1951). *Surg. Gynec. Obstet.*, **93**, 153.
Short, D. S. (1953). *Brit. Med. J.*, **i**, 790.
Sibthorpe, E. M. (1955). *Brit. Med. J.*, **ii**, 1163.
de Swiet, J. (1955). *Brit. Med. J.*, **ii**, 1253.
de Takats, G., Beck, W. C., Fenn, G. K. (1939). *Surgery*, **6**, 339.

## Figures and Tables

**Figure f1:**
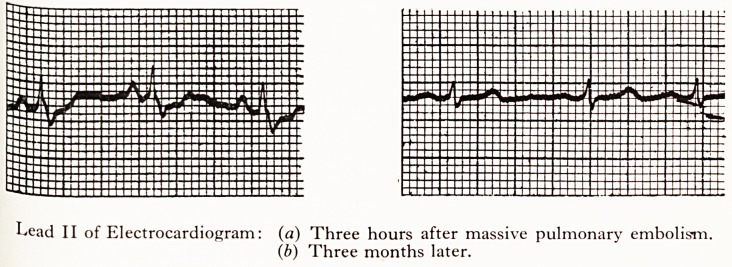


**Figure f2:**